# Poldip2 controls leukocyte infiltration into the ischemic brain by regulating focal adhesion kinase-mediated VCAM-1 induction

**DOI:** 10.1038/s41598-021-84987-z

**Published:** 2021-03-10

**Authors:** Lori N. Eidson, Qingzeng Gao, Hongyan Qu, Daniel S. Kikuchi, Ana Carolina P. Campos, Elizabeth A. Faidley, Yu-Yo Sun, Chia-Yi Kuan, Rosana L. Pagano, Bernard Lassègue, Malú G. Tansey, Kathy K. Griendling, Marina S. Hernandes

**Affiliations:** 1 Department of Physiology, Emory University, Atlanta, GA 30322 USA; 2 Division of Cardiology, Department of Medicine, Emory University, 101 Woodruff Circle, 308-C WMB, Atlanta, GA 30322 USA; 3 Department of Neuroscience, University of Virginia, Charlottesville, VA 22904 USA; 4 Department of Neuroscience, Hospital Sírio-Libanês, São Paulo, Brazil; 5 Department of Neuroscience, Center for Translational Research in Neurodegenerative Disease, College of Medicine, Normal Fixel Institute for Neurological Diseases, University of Florida, Gainesville, FL 32610 USA; 6 Department of Neurology, Center for Translational Research in Neurodegenerative Disease, College of Medicine, Normal Fixel Institute for Neurological Diseases, University of Florida, Gainesville, FL 32610 USA

**Keywords:** Cell signalling, Stroke, Blood-brain barrier

## Abstract

Stroke is a multiphasic process involving a direct ischemic brain injury which is then exacerbated by the influx of immune cells into the brain tissue. Activation of brain endothelial cells leads to the expression of adhesion molecules such vascular cell adhesion molecule 1 (VCAM-1) on endothelial cells, further increasing leukocyte recruitment. Polymerase δ-interacting protein 2 (Poldip2) promotes brain vascular inflammation and leukocyte recruitment via unknown mechanisms. This study aimed to define the role of Poldip2 in mediating vascular inflammation and leukocyte recruitment following cerebral ischemia. Cerebral ischemia was induced in Poldip2^+/+^ and Poldip2^+/−^ mice and brains were isolated and processed for flow cytometry or RT-PCR. Cultured rat brain microvascular endothelial cells were used to investigate the effect of Poldip2 depletion on focal adhesion kinase (FAK)-mediated VCAM-1 induction. Poldip2 depletion in vivo attenuated the infiltration of myeloid cells, inflammatory monocytes/macrophages and decreased the induction of adhesion molecules. Focusing on VCAM-1, we demonstrated mechanistically that FAK activation was a critical intermediary in Poldip2-mediated VCAM-1 induction. In conclusion, Poldip2 is an important mediator of endothelial dysfunction and leukocyte recruitment. Thus, Poldip2 could be a therapeutic target to improve morbidity following ischemic stroke.

## Introduction

Polymerase δ-interacting protein 2 (Poldip2, PDIP38, Mitogenin-1) is a ubiquitously expressed multifunctional protein first identified as a binding partner of polymerase δ and proliferating cell nuclear antigen^1^. While originally described to be involved in DNA replication and damage repair, subsequent studies have implicated Poldip2 as a critical regulator of focal adhesion turnover, mitochondrial function and morphology, cell migration, proliferation and differentiation, sensory nerve activation, autophagy and, more recently, metabolic adaptation^1,2^. Homozygous gene trap mice suffer from perinatal lethality^3^ and Poldip2 heterozygous mice have a clear vascular phenotype characterized by arterial stiffness, increased collagen deposition, disordered elastic laminae and impaired collateral formation^4,5^. Studies from our group have shown that Poldip2 heterozygous mice are strongly protected against blood–brain barrier (BBB) permeability and the expression of inflammatory cytokines that accompanies cerebral ischemia^6^ and sepsis-associated encephalopathy^7^. These studies, in addition to our report on the protective effect of Poldip2 depletion in a lipopolysaccharide (LPS) model of acute respiratory distress syndrome^8^, suggest that Poldip2 may play a critical role in regulating endothelial barrier function. However, whether or not a reduction in Poldip2 also influences leukocyte recruitment and transmigration across the BBB is unknown.

Adhesion molecules, such as ICAM-1, VCAM-1 and selectins, mediate the recruitment and firm adhesion of circulating leukocytes from the bloodstream to the postischemic cerebral microvasculature, facilitating their transmigration across the vascular endothelial barrier^9^. Leukocyte-endothelial cell adhesion, which is largely absent in the normal healthy cerebral microcirculation, becomes substantial and persists for hours and days following the onset of brain ischemia, compromising cerebral microvascular perfusion and contributing to the severity of stroke outcomes^9,10^.

Cytokines, reactive oxygen species (ROS), and prostaglandin E2 (PGE2) are some of a large number of inflammatory mediators released into ischemic brain tissue and plasma, activating transcription-dependent and -independent pathways, resulting in upregulation of cell adhesion molecules^9,11^. Patterns of adhesion molecule expression are very dynamic and are closely associated with the infiltration of different subsets of leukocytes. Evaluation of plasma levels of adhesion molecules in patients with acute ischemic stroke and histopathological examination of brain tissue in experimental stroke models revealed that expression of E-selectin, which is associated with neutrophil extravasation, peaks early (6–24 h) after acute cerebral ischemia and rapidly decreases thereafter, corresponding to the onset of neutrophil recruitment into the brain tissue^12,13^. VCAM-1, in turn, which predominantly mediates adhesion of monocytes, progressively increases and peaks 5–10 days after cerebral ischemia, corresponding to the onset of monocyte recruitment^12–15^. Because VCAM-1 mediates sustained and prolonged leukocyte adhesion and transmigration, there is an increasing appreciation for the role of VCAM-1 in coordinating the inflammatory response and leukocyte recruitment in cerebral ischemia.

In endothelial cells, transcriptional regulation of VCAM-1 involves AP-1, NF-κB and GATA-4 activation^16–18^. In addition, nuclear focal adhesion kinase (FAK) activity has been shown to be involved in promoting tumor necrosis factor (TNF)-induced VCAM-1 expression in human umbilical vein endothelial cells^19^. However, specific factors regulating FAK-induced VCAM-1 expression remain unclear and comparatively much less is known about the mechanisms that regulate inflammation-induced FAK activation. Of interest, Poldip2 has been shown to regulate FAK activity^20^.

Based on these considerations and our previous work implicating Poldip2 in endothelial barrier function, we hypothesized that heterozygous deletion of Poldip2 will diminish infiltration of leukocytes into the brain. We examined the effect on several different subtypes of leukocytes and investigated the mechanistic basis of the role of Poldip2 by testing the hypothesis that Poldip2 mediates TNF-induced VCAM-1 expression via regulation of FAK activity. Herein we report that Poldip2^+/−^ mice exhibit reduced infiltration of leukocytes into the brain, as well as lower VCAM-1 expression, following cerebral ischemia. Our data further suggest that Poldip2 depletion in cultured rat brain microvascular endothelial cells (RBMECs) prevents VCAM-1 induction, leukocyte adhesion and TNF-induced FAK activation. Overexpressing FAK partially rescues VCAM-1 expression induced by TNF in spite of Poldip2 depletion. Thus, we substantiated Poldip2 as an important mediator of leukocyte infiltration into the brain and identified FAK activation as a critical intermediary in Poldip2-mediated VCAM-1 induction.

## Methods

### Animals

Poldip2 gene trap mice on the C57BL/6 background were produced by the Texas A&M Institute for Genomic Medicine (College Station, TX). Mice were genotyped using a 4-primer qPCR method. Homozygous gene trap mice suffer from perinatal lethality; therefore, Poldip2^+/−^ mice were used for this study. Characterization of these mice has previously been described^3^. Studies involving animals were carried out in compliance with the ARRIVE guidelines^21,22^. All animal experiments were conducted with the approval of the Institutional Animal Care and Use Committee at Emory University (protocol reference number: 201700864). Ethical approval was received before conducting the study.

### Animal model of cerebral ischemia

Hypoxia-induced permanent stroke was induced in 10- to 14-week-old male mice, as previously described^23,24^. This stroke model leads to reperfusion deficits, fibrin and platelet deposition, and significant infarct in the middle cerebral artery-supplied territory^24^. Mice were anesthetized with isoflurane (medical air (21% oxygen) delivered at 0.5 l/min with 3% isoflurane for induction and 1.5% for maintenance). The right common carotid artery (RCCA) was exposed and transiently occluded, using 5-0 silk sutures to interrupt blood flow to the ipsilateral side of the brain. The wound was temporarily closed and animals were turned to a prone position. To induce transient hypoxia, medical air in the inhalation mixture was replaced with 7.5% oxygen balanced by nitrogen (92.5% N_2_) for 30 min. A rectal probe was connected to a temperature controller system with heating lamps and core body temperature was maintained at 37.5 ± 0.5 °C during the entire procedure. At the end of the hypoxic stimulation, mice were returned to their original supine position, the inhalation mixture was switched back to medical air and the RCCA sutures were released. Sham-operated control mice simply underwent exposure of the RCCA and were kept under anesthesia for the same duration as the others.

### Lesion volume

Twenty-four hours after cerebral ischemia induction, mice were deeply anesthetized and sacrificed by cervical dislocation. Brains were removed, sliced into 2-mm coronal sections, and the volume of the ischemic lesion was measured by 2,3,5-triphenyltetrazolium chloride (TTC) staining as described previously^6,25^. The lesion volume was calculated using ImageJ.

### Brain dissociation for immune cell isolation

Forty-eight hours after cerebral ischemia induction mice were anesthetized with isoflurane and sacrificed by cervical dislocation. Whole brains were immediately removed, finely minced in 1 × Hank’s balanced salt solution (HBSS, without calcium, magnesium, or phenol red; Invitrogen, Carlsbad, CA), transferred to an enzymatic solution in DMEM/F12 media containing 1 mg/ml papain (Sigma Aldrich, St. Louis, MO), 1.2 U/ml dispase II (Roche diagnostics, Risch-Rotkreuz, Switzerland), and 220 U/ml DNase I (Invitrogen) and incubated at 37 °C for 20 min with agitation every 5 min. Warm fetal bovine serum (10%; heat inactivated, Atlanta Biologicals) was added to neutralize the enzymatic reaction, and samples were spun at 300×*g* for 5 min. The supernatant was removed and the tissue pellet was homogenized in ice cold 1 × HBSS by pipetting up and down 30 times using a fine-tip (Thermo Fisher Scientific, Waltham, MA), fire-polished glass pipette. The homogenate was passed through a sterile 70 μM cell strainer and resuspended in 37% Percoll (Percoll pH 8.5–9.5; Sigma Aldrich). Four ml of 70% Percoll were layered below the tissue suspension, and 4 ml of 30% Percoll were overlain, followed by 4 ml of 1 × HBSS and spun at 500×*g* for 30 min at room temperature with no brake. The immune cell cloud was identified and collected between the 70% and 37% layers and washed with 4 volumes of 1 × HBSS. Cells were plated on a 96 well plate for immunocytochemistry.

### Multi-colored flow cytometry

All cells were stained with Live/Dead Fixable Aqua (1:2000; Invitrogen) at room temperature for 30 min in the dark. Following the Live/Dead viability stain, cells were incubated with anti-mouse CD16/CD32 (Fc block; 1:100, eBioscience), CD3 PE-eFluor610 (1:100; eBioscience), CD4 PE (1:100; eBioscience), CD8b APC-eFluor780 (1:100; eBioscience), CD11b PE-Cy7 (1:200; eBioscience), Ly6G Pacific Blue (1:100; BioLegend), CD45 PerCP-Cy5.5 (1:100; eBioscience), CD11c Alexa Fluor 700 (1:50; BioLegend) Ly6C FITC (1:200; eBioscience) in FACS buffer. Samples were run on an LSRII (BD Biosciences) flow cytometer and analyzed with FlowJo_V10 software.

Single cell lymphocytes were gated on forward scatter area (FSA; size) and side scatter area (SSA; granularity) and then by forward scatter height (FSH) and FSA. Live cells were selected as the negative Fixable Aqua population. CD45^+^ cells were selected and gated by CD3 (T cells) versus CD11b (myeloid cells). The CD3^+^ population was then gated on CD4 (helper T cells) and CD8 (cytotoxic T cells). CD11b^+^ cells were further gated on CD11c and Ly6G for the following populations: CD11c^−^Ly6G^−^ (microglia and macrophages), CD11c-Ly6G^+^ (neutrophils), and CD11c^+^Ly6G^−^ (dendritic cells). CD11c^−^Ly6G^−^ cells were gated for CD45 and low and high populations were further gated for MHCII and Ly6C to determine their activation state (Suppl. Fig. S1).

### RNA extraction and RT-qPCR

Total RNA was purified with the RNeasy Plus kit (Qiagen, Chatsworth, CA). Reverse transcription was performed using Protoscript II reverse transcriptase (New England Biolabs, Ipswich, MA) with random primers and cDNA was purified with the QIAquick kit (Qiagen). cDNA was amplified with primers against Glyceraldehyde 3-phosphate dehydrogenase (GAPDH) (5′-CTGGAGAAACCTGCCAAGTA-3′, 5′-TGTTGCTGTAGCCGTATTCA-3′), E-selectin (5′- AGCTATAATTCCTCCTGCTC-3′, 5′-GAGGAACATTTCCTGATACC-3′), P-selectin (5′-CTAGAGAGAGATTCCACGAG-3′, 5′-CTACTGAGGTTAGACTCCAC-3′), ICAM-1 (5′-CAAGGAGGACCTCAGCCTGG-3′, 5′-GGTGAGGTCCTTGCCTACTT-3′) and VCAM-1 (5′-GAGTGTACAGCCTCTTTATG-3′, 5′- CTGCAGTTCCCCATTATTTAG-3′) using an annealing temperature of 55 °C and Biotium 2X Forget-Me-Not EvaGreen qPCR Master Mix with Low ROX (Biotium, Fremont, CA). Reactions were carried out in 96 wells qPCR plates, using a QuantStudio 7 Flex System (Thermo Fisher Scientific, Waltham, MA) Real-Time qPCR System. Data analysis was performed using the mak3i module of the qpcR software library^26,27^ in the R environment^28^.

### Cell culture

Primary RBMECs (CellBiologics; Cat No. RA-6023) were cultured in endothelial cell medium (CellBiologics) supplemented with 2% fetal bovine serum, endothelial cell growth factors, and antibiotics (CellBiologics; Cat No. M1266-Kit). Media was changed every 2 days until cells reached confluence. All experiments were conducted between passages 4–6. In each experiment, cultures exposed to TNF for 6 h (10 ng/ml; Thermo Fisher Scientific) were compared with PBS control conditions. The FAK inhibitor, PF-562,271 (FAK-I; 0.5–2.5 μM Sigma Aldrich, St. Louis, MO) was added to RBMECs for 1 h before TNF addition for 6 h.

### Small interfering RNA

70–80% confluent RBMECs were transfected with either rat siPoldip2 (sense 5′-GUCUAUUGGUGGCGAUACU[dT][dT]-3′ antisense 5′-AGUAUCGCCACCAAUAGAC [dT][dT]-3′; Sigma), rat siVCAM-1 (sense 5′-CCAGAUAGACAGUCCACUA[dT][dT]-3′ antisense 5′- UAGUGGACUGUCUAUCUGG [dT][dT]-3′; Sigma) or control siRNA (MISSION siRNA Universal Negative Control #1; Sigma; Cat No. SIC001). Cells were washed with HBSS and transfected with 100 nM of siRNA and Lipofectamine RNAiMAX Reagent (volume equal to double the volume of siRNA; Invitrogen; Cat No. 13778150) in Opti-MEM reduced serum media (Gibco; Cat No. 31985-070). Cells were incubated with siPoldip2 for 5 h or with siVCAM-1 for 12 h, and Opti-MEM was replaced with complete culture medium for an additional 48 h until cells were exposed to TNF for 6 h. Gene silencing was confirmed by immunoblotting.

### Adenoviruses

Adenoviruses expressing FAK (AdFAK), LacZ control (AdLacZ, control for AdFAK) and coexpressing human Poldip2-myc (AdPoldip2) and green fluorescent protein (GFP) or GFP alone (AdGFP) were prepared in HEK293T cells as described previously and precipitated with polyethylene glycol 6000 (PEG-6000)^29^. AdLacZ was a kind gift of Dr. Aviv Hassid (University of Tennessee) and the AdFAK was a kind gift from Dr. Joseph C. Loftus (Mayo Clinic, Phoenix, AZ). RBMECs were transduced with recombinant adenoviruses for 3 h at 37 °C in complete endothelial cell medium. Following 3 h, fresh media was added to RBMECs and cells were allowed to recover for 24 h. Cells were then washed with HBSS and fresh media was added for an additional 24 h before use in experiments. Overexpression of FAK and Poldip2 was confirmed by immunoblotting.

### Western blotting

Whole cell lysate was prepared from cultured RBMECs using a buffer containing 50 mM HEPES, 50 mM NaCl, 5 mM EDTA, 10 µg/ml aprotinin, 10 µg/ml leupeptin, 1 mM PMSF, and Halt phosphatase inhibitor cocktail (Thermo Fisher Scientific). Homogenates were centrifuged at 17,700×*g* at 4 °C for 2 min and then sonicated for 10 s. Homogenates were aliquoted, and samples were brought to equal volume in Laemmli buffer and water before boiling for 10 min at 100 °C. Samples were stored at − 20 °C until gel loading. Following separation by SDS–PAGE, proteins were transferred to PVDF membranes with 0.45 μm pore size (Cat No. IPVH00010; Milipore) and assessed by western blotting with primary antibodies against Poldip2 (ab181841; Abcam), VCAM-1 (ab134047; Abcam), FAKpY397 (611806; BD Bioscience), total FAK (06-543; EMD Millipore) and β-tubulin (A5441; Sigma). Blots were incubated with horseradish peroxidase (HRP)-conjugated secondary antibodies [anti-mouse (NA931; GE) or anti-rabbit (70745; Cell Signaling)] and assessed using enhanced chemiluminescence (ECL, Thermo Fisher Scientific). HRP-induced luminescence was detected with Amersham Hyperfilm ECL (GE). Detected bands were scanned and densitometry was performed using ImageJ.

### THP-1 adhesion assay

Human monocytes (THP-1 cell line) were spun down at 1500 × rpm, 5 min, 20 ºC and resuspended in serum-free Dulbecco’s modified Eagle’s medium (DMEM, Sigma, D5671) supplemented with 0.2% bovine serum albumin (BSA, Sigma, Cat No. 3117332001), 2 mM L-glutamine (ThermoFisher, Cat No. 25030), penicillin and streptomycin. THP-1 cells were labeled with Hoechst 33342 (5 µg/ml-ThermoFisher, Cat No. 62249) at 37 ºC for 10 min. Cells were then spun down, washed with DMEM, re-spun and then re-suspended in fresh DMEM with 0.2% BSA (7 × 10^4^ cells per ml). Monolayers of RBMECs were transfected with siControl or siPoldip2 and, after 48 h stimulated with PBS or TNF (10 ng/ml) for 6 h prior to the adhesion assay. For adhesion assays in which Poldip2 was overexpressed, RBMECs were transduced with viruses expressing AdPoldip2 or AdGFP as described above and kept in regular media for 48 h. Cells were then transfected with siControl or siVCAM-1 and, after 48 h stimulated with PBS or TNF (10 ng/ml) for 6 h prior to the adhesion assay. RBMECs were then washed once with 0.2% BSA DMEM followed by addition of THP-1 cells (3.5 × 10^4^ cells per well, 24-well plate). THP-1 and RBMECs were incubated together for 30 min at 37 ºC. Cells were then washed twice with warm PBS followed by fixation in 3.7% paraformaldehyde (Electron Microscopy Science, Cat No. 15714-S) for 10 min at room temperature. Fixed cells were washed once in PBS and immediately imaged using a 10 × objective lens. Images were acquired on an Olympus IX71 Inverted fluorescent microscope with a DAPI fluorescence filter. Three representative pictures were acquired per sample. Images were imported using ImageJ. Background was subtracted from images and an image threshold was generated. Stained THP-1 nuclei were counted using the analyze particles function to evaluate adhesion to RBMECs.

### Statistical analysis

Data are expressed as mean ± SEM. Statistical analyses were performed using 2-way ANOVA with a Tukey’s multiple comparison test. For E-selectin RT-qPCR and Poldip2 overexpression western blotting, significance was determined using an unpaired t-test. All calculations were performed using GraphPad PRISM (version 7.04). *p* < 0.05 was considered statistically significant.

### Ethics approval and consent to participate

No human data or tissues were used in this study. This study was carried out in strict accordance with the recommendations in the Guide for the Care and Use of Laboratory Animals of the National Institutes of Health. Adequate measures were taken to minimize pain and suffering. This study was carried out in compliance with the ARRIVE guidelines and all animal welfare and experimental protocols were approved by the Emory University Institutional Animal Care and Use Committee (Protocol number: 201700864).

### Consent for publication

All contributing authors have given their consent for the publication of this study.

## Results

### Poldip2 regulates the recruitment of immune cells into brain tissue after cerebral ischemia

**Figure 1:**
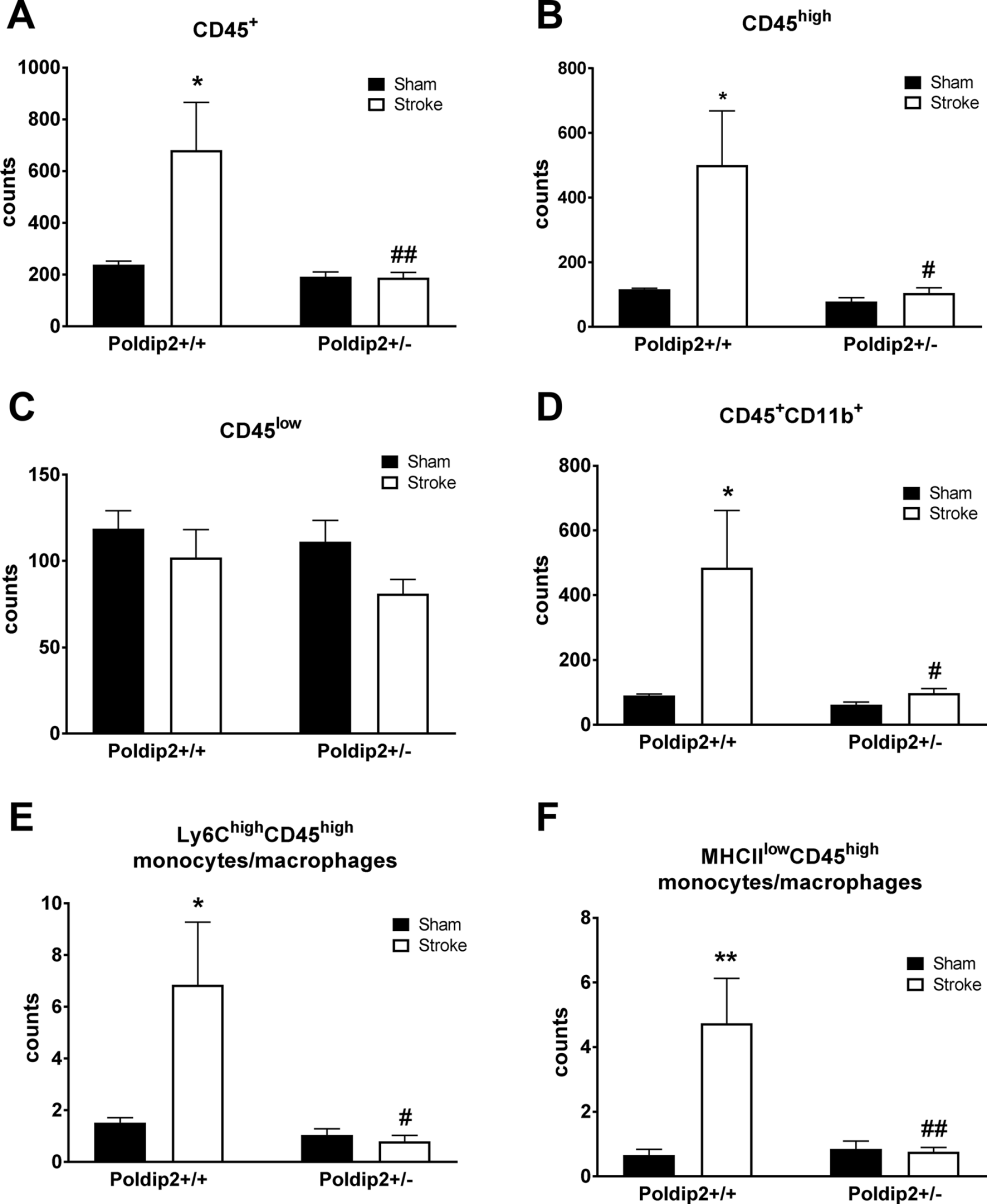
Poldip2 regulates the recruitment of leukocytes following cerebral ischemia induction. (A–F) Major brain immune subsets were characterized using quantitative multi-colored flow cytometry 48 h after sham surgery or hypoxia-induced permanent stroke in Poldip2^+/+^ and ^+/−^ mice. Bar graphs represent means ± SEM counts from 6–8 mice per group adjusted using counting beads. Two-way ANOVA *P < 0.05, **P < 0.01 vs. Poldip2^+/+^ sham mice, and ^#^P < 0.05, ^##^P < 0.01 vs. Poldip2^+/+^ ischemic mice.

Quantitative flow cytometric analysis of CD45 expression, a leukocyte common antigen, performed 48 h after cerebral ischemia induction, demonstrated that the number of CD45^+^ cells was dramatically increased in the brains of Poldip2^+/+^, but not in Poldip2^+/−^ mice (Fig. 1). Similar results were found for the CD45^high^ immune cell population. Additionally, the infiltration of CD45^+^CD11b^+^ myeloid cells, Ly6C^high^CD45^high^ and MHCII^low^CD45^high^ inflammatory monocytes/macrophages was significantly reduced in Poldip2^+/−^ mice after cerebral ischemia when compared to Poldip2^+/+^ mice. CD45^low^ inflammatory cells were not significantly increased in the brains of either Poldip2^+/+^ or Poldip2^+/−^ mice (Fig. 1). Further characterization of invading cell populations demonstrated that CD3^+^, CD8^+^ and CD4^+^ T cells and CD11b^+^Ly6G^−^ dendritic cell counts were not significantly increased in the brains of either Poldip2^+/+^ or Poldip2^+/−^ mice 48 h after cerebral ischemia (Suppl. Fig. S2). Similar results were found for CD11b^+^Ly6G^+^ neutrophils (Poldip2^+/+^: 8.3 ± 8.4 vs. Poldip2^+/−^: 2.3 ± 1.5, p = 0.17), Ly6C^low^CD45^high^ (Poldip2^+/+^: 3.5 ± 3.7 vs. Poldip2^+/−^: 0.3 ± 0.3, p = 0.06 and MHCII^high^CD45^high^ (Poldip2^+/+^: 9.9 ± 12.5 vs. Poldip2^+/−^: 0.8 ± 0.9, p = 0.15) (data not shown).

### Poldip2 does not affect lesion volume after cerebral ischemia induction

We then tested whether Poldip2 depletion affects the lesion volume following cerebral ischemia induction. Brains obtained from Poldip2^+/+^ and Poldip2^+/−^ mice were analyzed using TTC staining. Twenty-four hours after cerebral ischemia induction, no significant differences were observed between Poldip2^+/+^ and Poldip2^+/−^ mice (Suppl. Fig. S3).

### Heterozygous deletion of Poldip2 reduces leukocyte adhesion molecule expression in vivo

**Figure 2:**
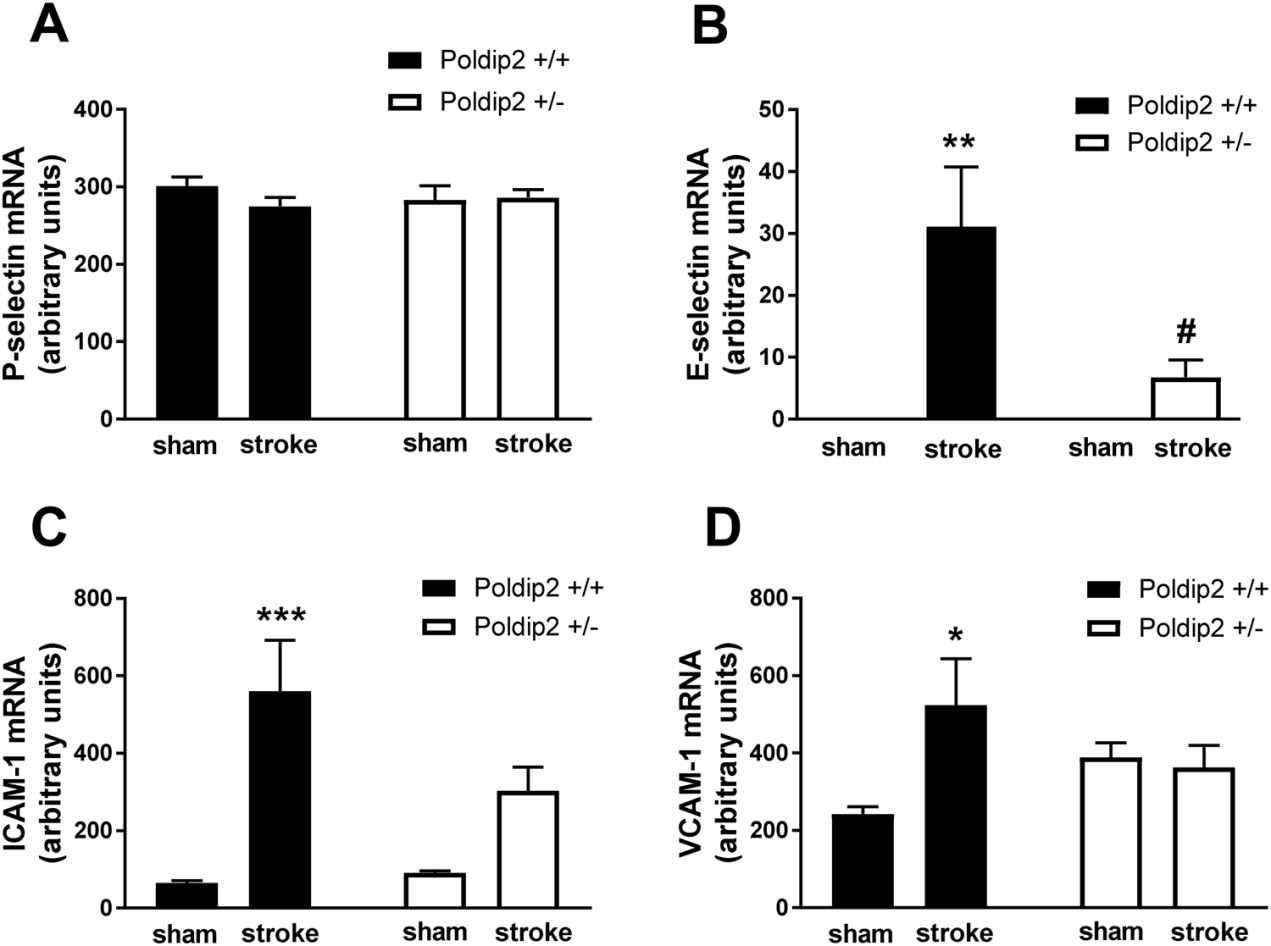
Poldip2 depletion reduces the induction of leukocyte adhesion molecules in vivo. P-selectin (**A**), E-selectin (**B**), ICAM-1 (**C**) and VCAM-1 (**D**) and mRNAs were measured by RT-PCR in the brain tissue 24 h after sham surgery or hypoxia-induced permanent stroke in Poldip2^+/+^ and ^+/−^ mice. Bar graphs represent means ± SEM from 5 to 9 mice per group normalized to GAPDH. Two-way ANOVA *P < 0.05, **P < 0.01, ***P < 0.001 vs. Poldip2^+/+^ sham mice and # P < 0.05 vs. Poldip2^+/+^ ischemic mice.

Following cerebral ischemia, increased expression of adhesion molecules in cerebral endothelial cells promotes the recruitment of circulating leukocytes into brain tissue. To determine whether Poldip2 regulates the expression of leukocyte adhesion molecules, brains from Poldip2^+/+^ and Poldip2^+/−^ mice were harvested 24 h after cerebral ischemia induction and E-selectin, P-selectin, ICAM-1 and VCAM-1 mRNA levels were measured. P-selectin mRNA levels remained unchanged in the brain tissue upon cerebral ischemia induction in both Poldip2^+/+^ and Poldip2^+/−^ mice. While E-selectin expression was not detectable in sham samples, E-selectin mRNA levels were increased 24 h after ischemia, but significantly less so in Poldip2^+/−^, compared to Poldip2^+/+^ mice. Similarly, ICAM-1 and VCAM-1 mRNA levels were increased in the brain tissue of Poldip2^+/+^ mice after ischemia, but not in Poldip2^+/−^ mice (Fig. 2).

### Poldip2 silencing attenuates TNF-induced VCAM-1 upregulation and leukocyte adhesion to brain endothelial cells in vitro

**Figure 3:**
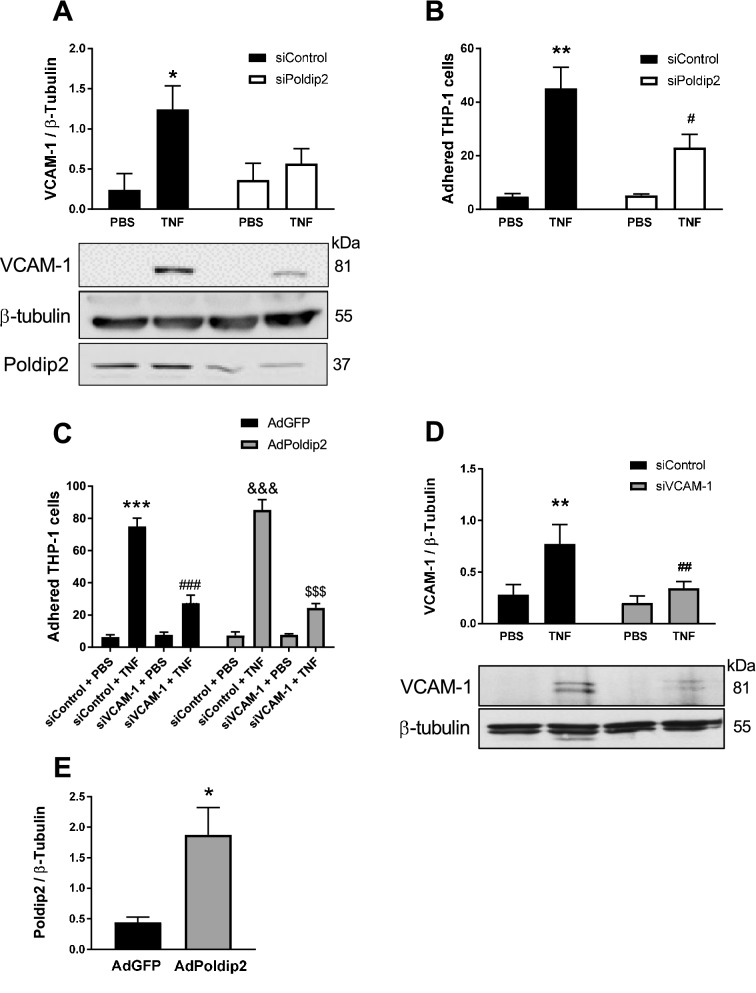
Poldip2 mediates VCAM-1 expression in RBMECs. (**A**) Confluent RBMECs were transfected with siControl or siPoldip2 before exposure to PBS or TNF (10 ng/ml) for 6 h. VCAM-1 protein expression was measured by western blotting and densitometry. Down-regulation of Poldip2 after siRNA transfection was verified by western blotting. Bars represent means ± SEM of 4–5 independent experiments normalized to β-tubulin. Two-way ANOVA *P < 0.05 vs. siControl PBS. (**B**) Adhesion of THP-1 monocytes to RBMECs was determined by light microscopy. Bars represent means ± SEM of 3 independent experiments. Two-way ANOVA **P < 0.01 vs siControl PBS and ^#^P < 0.05 vs siControl TNF. (**C**) Seventy-80% confluent RBMECs were transduced with AdGFP or AdPoldip2 and kept in regular media for 48 h. Cells were then transfected with siControl or siVCAM-1 and, after 48 h stimulated with PBS or TNF (10 ng/ml) for 6 h prior to the adhesion assay. Bars represent means ± SEM of 5 independent experiments. Two-way ANOVA ***P < 0.001 vs AdGFP siControl PBS, ^&&&^P < 0.001 vs AdPoldip2 siControl PBS, ^###^P < 0.001 vs AdGFP siControl TNF and ^$$$^P < 0.001 vs AdPoldip2 siControl TNF. (**D**) Down-regulation of VCAM-1 after siRNA knockdown was verified by western blotting. Bars represent means ± SEM of 3 independent experiments. Two-way ANOVA **P < 0.01 vs siControl PBS and ^##^P < 0.01 vs siControl TNF. (**E**) Overexpression of Poldip2 was verified by western blotting. Bars represent means ± SEM of 4 independent experiments. t-test *P < 0.05.

Longitudinal studies evaluating the levels of soluble adhesion molecules in human peripheral blood after cerebral ischemia revealed that while ICAM-1 and E-selectin levels peaked within the first 6–24 h and rapidly decreased thereafter, VCAM-1 levels progressively increased and peaked after 5–10 days, suggesting that VCAM-1 plays an important role mediating prolonged and sustained leukocyte adhesion and migration in cerebral ischemia^13–15^. We have previously determined that Poldip2 is required for LPS-induced VCAM-1 expression in human primary pulmonary microvascular endothelial cells^8^. To examine the role of Poldip2 in the regulation of VCAM-1 induction in the brain, cultured RBMECs were transfected with siPoldip2 or control siRNA (siControl) and treated with TNF (10 ng/ml) for 6 h before evaluating VCAM-1 expression. TNF was used to stimulate RBMECs because it is one of the main proinflammatory cytokines implicated in the vascular permeability induced by stroke^30,31^ and because it is a key factor regulating inflammatory VCAM-1 expression^19^. Consistent with our in vivo observation, Poldip2 silencing significantly inhibited TNF-induced VCAM-1 upregulation. Poldip2 silencing efficiency was evaluated by western blotting (Fig. 3A). The physiological consequence of VCAM-1 reduction in RBMECs was assessed by evaluating THP-1 monocyte adherence. RBMECs were transfected with siControl or siPoldip2, stimulated with TNF (10 ng/ml) for 6 h and then incubated with THP-1 monocytes. Poldip2 downregulation in RBMECs significantly decreased TNF-induced THP-1 adherence (Fig. 3B), suggesting that Poldip2 in endothelial cells may play a role in regulating leukocyte adhesion during cerebral ischemia. To determine if VCAM-1 is required for Poldip2-mediated effects on monocyte adhesion, we examined the ability of Poldip2 to mediate THP-1 adhesion upon VCAM-1 depletion. TNF (10 ng/ml, for 6 h) significantly enhanced THP-1 adhesion in RBMECs transfected with siControl compared with cells treated with PBS, regardless of whether Poldip2 was also overexpressed. However, in both control and Poldip2 overexpressing RBMECs, depletion of VCAM-1 using siRNA almost completely abolished THP-1 adhesion, indicating that VCAM-1 is required for monocyte adhesion in both control and Poldip2-overexpressing situations (Fig. 3C). VCAM-1 gene silencing (Fig. 3D) and Poldip2 overexpression (Fig. 3E) were confirmed using immunoblotting.

### Poldip2 silencing blocks TNF- induced FAK phosphorylation

**Figure 4:**
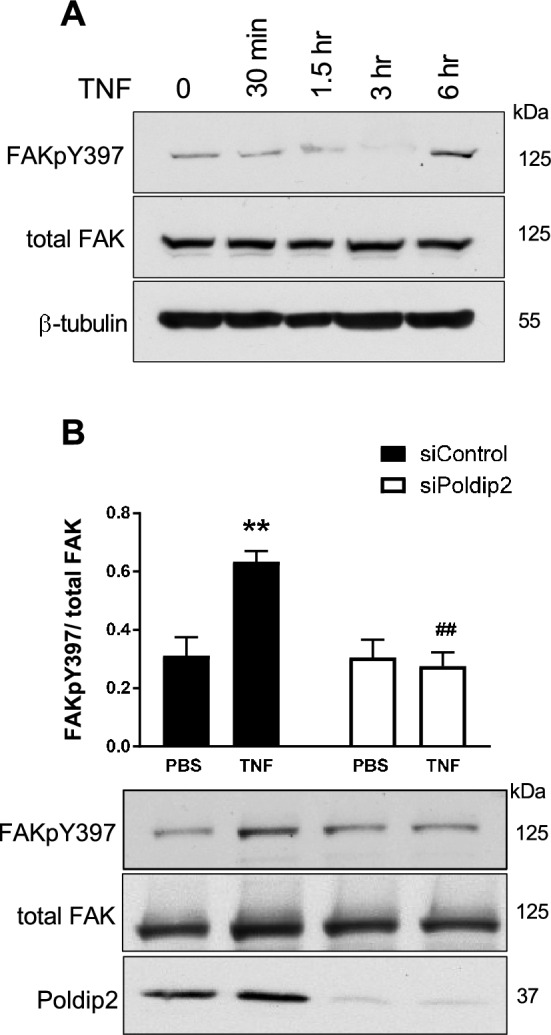
Poldip2 mediates TNF-induced FAK phosphorylation. (**A**) Confluent RBMECs were exposed to TNF (10 ng/ml) for 30 min, 1.5, 3 and 6 h. FAK phosphorylation on Y397 (FAKpY397), total FAK and β-tubulin protein levels were evaluated by western blotting. (**B**) Confluent RBMECs were transfected with siControl or siPoldip2 before exposure to PBS or TNF (10 ng/ml) for 6 h. FAKpY397, total FAK and β-tubulin protein expression were measured by western blotting and densitometry. Down-regulation of Poldip2 after siRNA knockdown was verified by western blotting. Bars represent means ± SEM of 3–5 independent experiments normalized to total FAK. Two-way ANOVA **P < 0.01 vs. siControl PBS and ^##^P < 0.01 vs. siControl TNF.

TNF induces VCAM-1 expression via several intracellular signaling pathways, which include MAPK, AP-1 and NF-κB^32^. Additionally, FAK signaling has been previously shown to facilitate TNF-induced VCAM-1 expression in HUVECs^19^. We have previously reported that Poldip2 regulates FAK activation in smooth muscle cells^20^. To first determine whether TNF induces FAK activation in RBMECs, cells were stimulated with TNF (10 ng/ml) for either 30 min, 1.5, 3 and 6 h and FAK phosphorylation on Y397 (FAKpY397) was assessed by western blotting assay. While total FAK protein levels remained unchanged, FAK phosphorylation was increased in response to 6 h of TNF treatment (Fig. 4A).

To test the hypothesis that Poldip2 also mediates FAK phosphorylation in cultured RBMECs, cells with Poldip2 depletion were treated with TNF (10 ng/ml for 6 h) and FAKpY397 phosphorylation was assessed by western blotting assay (Fig. 4B). Consistent with our previous findings, Poldip2 depletion abrogated TNF-induced FAK phosphorylation in RBMCEs. This result suggests Poldip2 is required for TNF-mediated FAK phosphorylation.

### FAK is required for TNF- induced VCAM-1 expression in vitro

**Figure 5:**
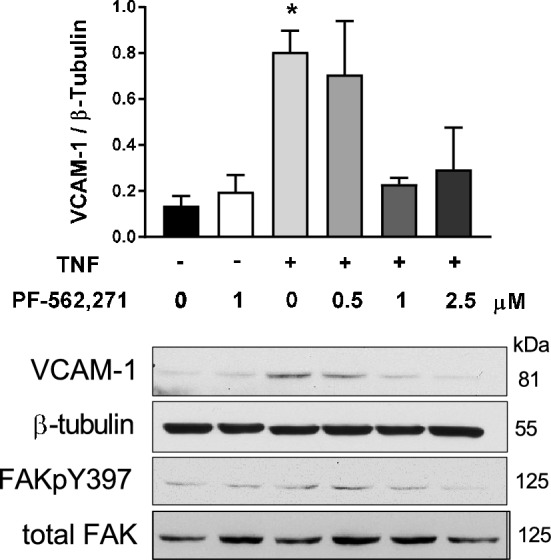
FAK activity mediates TNF-induced VCAM-1 upregulation. Confluent RBMECs were treated with vehicle or PF-562,271 (0.5–2.5 μM) for 1 h before cells were exposed to TNF (10 ng/ml) for 6 h. VCAM-1, FAKpY397, total FAK and β-tubulin protein levels were evaluated by western blotting. One-way ANOVA *P < 0.05 vs. no TNF and no PF-562,271.

We next sought to determine whether FAK is required for TNF induction of VCAM-1 in RBMECs. Monolayers of RBMECs were exposed to increasing concentrations (0.5–2.5 µM) of the FAK inhibitor, PF-562,271 for one hour before co-treatment with TNF for 6 h. VCAM-1, FAKpY397 and total FAK levels were assessed by western blotting. TNF-induced VCAM-1 expression was attenuated in response to FAK-I, reaching a maximum effect at 1 µM (Fig. 5). This result suggests FAK activity is required for TNF-mediated VCAM-1 expression.

### Poldip2 regulates FAK-mediated VCAM-1 induction

**Figure 6:**
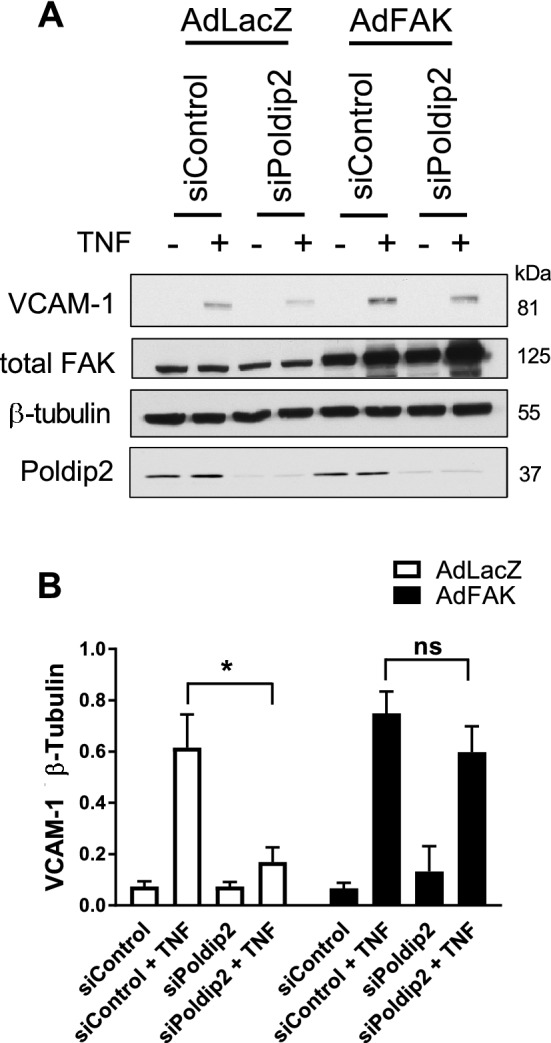
FAK activity mediates TNF-induced VCAM-1 upregulation. Seventy-80% confluent RBMECs were transfected with siControl or siPoldip2 and the following day transduced with AdFAK or AdLacZ (control for AdFAK). Two days after transduction, when cells had reached 100% confluence, RBMECs were stimulated with TNF (10 ng/ml). Six hours later, whole cell lysates were prepared and immunoblotted for VCAM-1, total FAK, Poldip2 and β-tubulin. Down-regulation of Poldip2 after siRNA knockdown as well as overexpression of FAK were verified by western blotting. (**A**) Representative VCAM-1, total FAK, Poldip2 and β-tubulin western blots. (**B**) Bars represent means ± SEM of 4–5 independent experiments normalized to β-tubulin. Two-way ANOVA *P < 0.05 vs. AdLacZ siPoldip2 TNF. *ns* not significant.

Our results suggest that Poldip2 is required for TNF-mediated activation of FAK (Fig. 4B) and induction of VCAM-1 (Fig. 3). Because TNF induction of VCAM-1 requires FAK (Fig. 5), we then hypothesized that FAK-mediated VCAM-1 expression is regulated by Poldip2. To test this hypothesis, we knocked down Poldip2 in the presence (AdFAK) or absence (AdLacZ) of FAK overexpression and measured TNF-induced VCAM-1 expression in RBMECS. Consistent with our earlier results, Poldip2 silencing attenuated TNF-induced VCAM-1 expression in cells infected with AdLacZ. In contrast, TNF-induced VCAM-1 expression was rescued in siPoldip2 RBMCEs overexpressing FAK (Fig. 6).

## Discussion

We previously reported that Poldip2 depletion in vivo protects against BBB permeability in the ischemic brain and improves survival^33^. In this study, we build upon our earlier work and report a novel mechanism by which Poldip2 regulates vascular inflammation and leukocyte recruitment into the ischemic brain. Notably, we observed that Poldip2 depletion in vivo blocked infiltration of CD45^+^, CD45^high^ immune cells, CD11b^+^CD45^+^ myeloid cells, as well as Ly6C^high^CD45^high^ and MHCII^low^CD45^high^ inflammatory monocytes/macrophages. We also show that Poldip2 depletion attenuated the induction of adhesion molecules such as E-selectin, ICAM-1 and VCAM-1. Focusing on VCAM-1, we demonstrate mechanistically that Poldip2 is required for both TNF-induced FAK activation and downstream induction of VCAM-1.

Stroke is a multiphasic process involving direct ischemic brain injury exacerbated by a secondary insult associated with the subsequent inflammatory response. Rapid activation of resident microglia and the production of proinflammatory cytokines, including TNF and IL-6, occur within hours of cerebral ischemia onset^34^. Inflammatory cytokines, in turn, upregulate adhesion molecules in endothelial cells, promoting transmigration of leukocytes across the BBB and post-ischemic neuroinflammation. Inhibition of leukocyte adhesion and transmigration across the BBB following cerebral ischemia reduces neuronal damage and improves functional outcome^35,36^. Therefore, elucidation of the molecular mechanisms regulating infiltration of inflammatory cells into ischemic tissue remains critical for the identification of potential pharmacological targets aimed at improving stroke outcomes.

Previous work from our group has demonstrated that Poldip2 depletion attenuates TNF and IL-6 levels following ischemic stroke, possibly via regulation of NFκB^33^. Both activation of brain resident immune cells and infiltration of blood-derived inflammatory cells contribute to the inflammatory reaction triggered by cerebral ischemia. Given the reduced levels of inflammatory markers in Poldip2^+/−^ mice^6^, in this report we investigated the contribution of Poldip2 to leukocyte recruitment into brain tissue after cerebral ischemia. Our results show an overall reduction in CD45^+^, CD45^high^ lymphocyte, CD11b^+^CD45^+^ myeloid cell, as well as Ly6C^high^CD45^high^ and MHCII^low^CD45^high^ inflammatory monocyte/macrophage in brains of Poldip2^+/−^ mice 48 h after cerebral ischemia induction. To investigate how Poldip2 depletion attenuates leukocyte recruitment, the mRNA levels of adhesion molecules were measured. ICAM-1, VCAM-1, and E-selectin mRNA levels were significantly attenuated in Poldip2^+/−^ animals following ischemic stroke compared to sham controls. Likewise, our in vitro studies demonstrated that depletion of Poldip2 attenuates TNF-induced VCAM-1 expression in RBMECs. While future work will be necessary to determine how Poldip2 regulates ICAM-1 and E-selectin, we focused here on determining the mechanism by which Poldip2 regulates VCAM-1.

TNF induces VCAM-1 expression via activation of the MAPK/FAK signaling pathways, which ultimately results in transcription factors, including AP-1, GATA-binding proteins and NFκB, binding to the VCAM-1 promoter^19^. In vascular smooth muscle cells, depletion of Poldip2 reduces FAK Y397 phosphorylation^20^, which. Raised the question of whether Poldip2 regulates FAK activation in RBMECs. Consistent with earlier findings, depletion of Poldip2 in RBMECs blocks TNF activation of FAKpY397. Additionally, our in vitro studies using a FAK inhibitor, PF-562,271, suggest that FAK is required for TNF-induced VCAM-1 expression in RBMECs. Together, our results suggest Poldip2 regulates TNF-mediated VCAM-1 expression via FAK activation, thus providing a potential mechanism by which Poldip2 mediates leukocyte infiltration, as shown in our in vivo flow cytometry results. Since FAK has also been described to regulate ICAM-1 induction, it is possible that Poldip2 enhances FAK activity to upregulate ICAM-1^37^. Future studies should investigate this potential mechanism.

Our data demonstrate that overexpression of FAK in TNF-treated cells with Poldip2 depletion largely, but not completely, recovers VCAM-1 expression (reduction of 68% between siControl + TNF and siPoldip2 + TNF in the presence of AdLacZ control virus vs*.* reduction of only 20% between siControl + TNF and siPoldip2 + TNF in the presence of AdFAK virus), suggesting the regulation of TNF-mediated VCAM-1 expression by Poldip2 may involve additional pathways. These additional pathways likely include NFκB, as Poldip2 depletion has previously been reported to inhibit NFκB activation and induction of downstream inflammatory effectors^7,33^. Interestingly, VCAM-1 induction in human pulmonary microvascular ECs was partially attenuated when cells were pre-treated with the mitochondrial ROS scavenger MitoTEMPO, which is consistent with the idea that additional mechanisms, such as FAK and NFκB, may be involved^8^.

Moreover, the specific downstream consequences of FAK activation by Poldip2 in RBMECs remain unknown. As described above, FAK-mediated VCAM-1 induction is associated with the activation of at least two different signaling pathways that involve the transcription factors AP-1 and GATA. Thus, Poldip2-induced FAK activation may affect these VCAM-1 transcription factors as well. Future research should investigate this potential regulation mechanism.

## Conclusions

In the context of our previous reports^7,8,33^, our findings provide additional evidence that Poldip2 is a critical regulator of endothelial barrier function and the inflammatory response through its modulation of adhesion molecule expression. Further work is needed to explore the role of Poldip2 in leukocyte activation per se as well as the upstream regulators of Poldip2. Future investigation should also focus on further determining how Poldip2 regulates FAK activation. Ultimately, Poldip2 inhibition may provide therapeutic benefit in a wide spectrum of brain pathologies involving barrier dysfunction.

## Supplementary Information

Supplementary Information

## Data Availability

All data generated or analyzed during this study are included in this article. The datasets used and/or analyzed during the current study are available from the corresponding author on reasonable request.

## Abbreviations

AP-1: Activator protein 1
BBB: Blood–brain barrier
BSA: Bovine serum albumin
ECL: Enhanced chemiluminescence
FAK: Focal adhesion kinase
FAK-I: Focal adhesion kinase inhibitor
FSA: Forward scatter area
FSH: Forward scatter height
GAPDH: Glyceraldehyde 3-phosphate dehydrogenase
HBSS: Hanks’ balanced salt solution
HRP: Horseradish peroxidase
ICAM-1: Intercellular adhesion molecule-1
IL-6: Interleukin 6
LPS: Lipopolysaccharide
MCP-1: Monocyte chemotactic protein-1
MAPK: Mitogen-activated protein kinase
NFκB: Nuclear factor kappa B
Poldip2: Polymerase (DNA-directed) δ-interacting protein 2
PGE2: Prostaglandin E2
qPCR: Quantitative polymerase chain reaction
RT-qPCR: Real-time quantitative polymerase chain reaction
RBMEC: Primary rat brain microvascular endothelial cells
ROS: Reactive oxygen species
RCCA: Right common carotid artery
siControl: Negative control small interfering RNA
siRNA: Small interfering RNA
THP-1: Human monocyte cell line
SSA: Side scatter area
TNF: Tumor necrosis factor
VCAM-1: Vascular cell adhesion molecule-1
