# Supplementary material for: Poldip2 controls leukocyte infiltration into the ischemic brain by regulating focal adhesion kinase-mediated VCAM-1 induction
Source: Sci Rep. 2021 Mar 10;11:5533. doi: 10.1038/s41598-021-84987-z (PMC7970934; doi:10.1038/s41598-021-84987-z)

Poldip2 controls leukocyte infiltration into the ischemic brain by regulating focal adhesion kinase-mediated VCAM-1 induction

Lori N. Eidson [1], Qingzeng Gao [2], Hongyan Qu [2], Daniel S. Kikuchi [2], Ana Carolina P. Campos [2,4], Elizabeth A. Faidley [2], Yu-Yo Sun [3], Chia-Yi Kuan [3], Rosana L. Pagano [4], Bernard Lassègue [2], Malú G. Tansey [1,5], Kathy K. Griendling [2], & Marina S. Hernandes [2*]

[1]Department of Physiology, Emory University, Atlanta GA 30322; [2]Division of Cardiology, Department of Medicine, Emory University, Atlanta GA 30322; [3]Department of Neuroscience, University of Virginia, Charlottesville, VA 22904; [4]Department of Neuroscience, Hospital Sírio-Libanês, São Paulo, Brazil; [5]Departments of Neuroscience and Neurology, Center for Translational Research in Neurodegenerative Disease, College of Medicine, Normal Fixel Institute for Neurological Diseases, University of Florida, Gainesville, FL 32610;

* Corresponding author: mshern2@emory.edu

Supplemental Figure 1

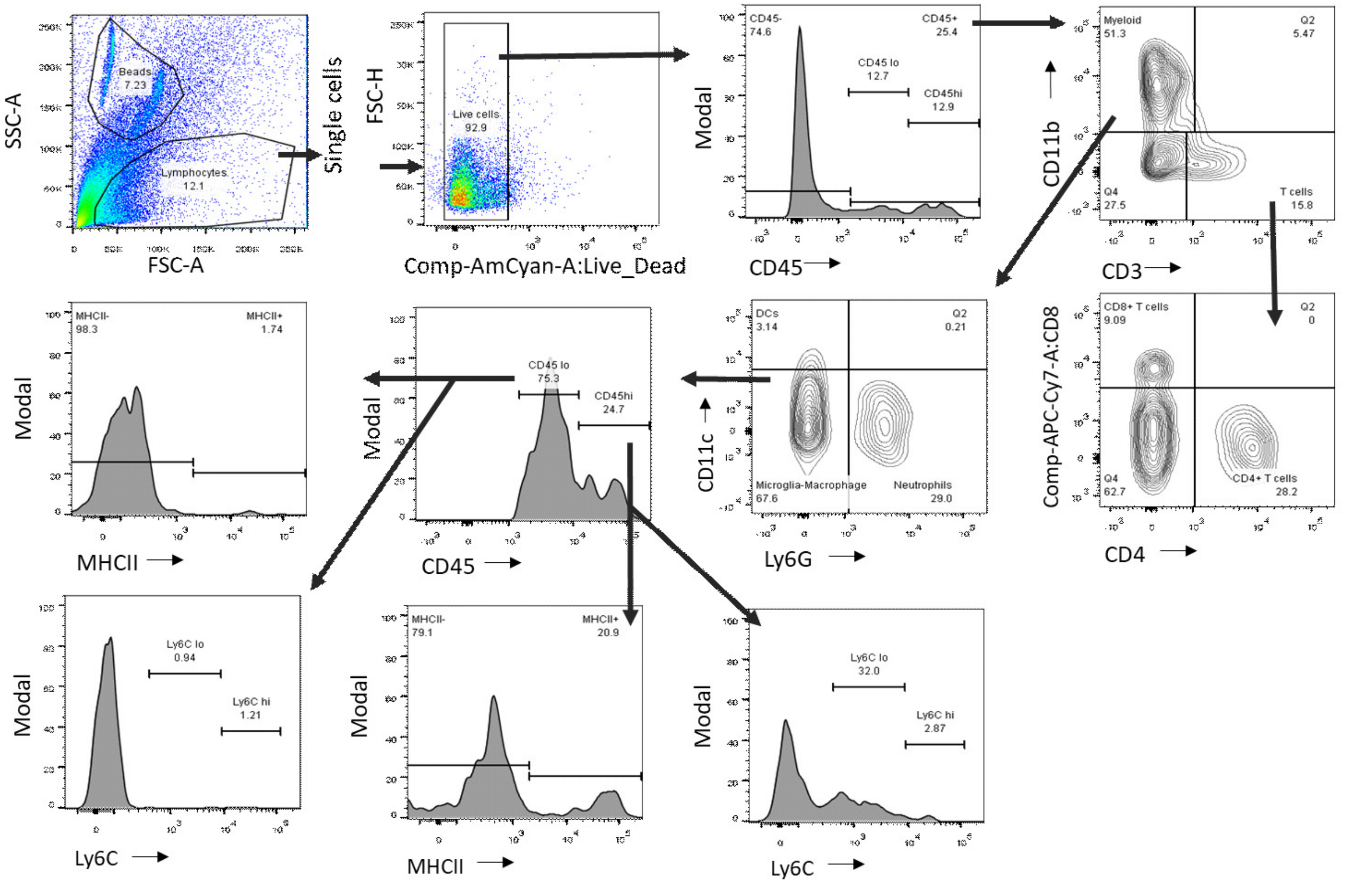

Supplemental Figure 2

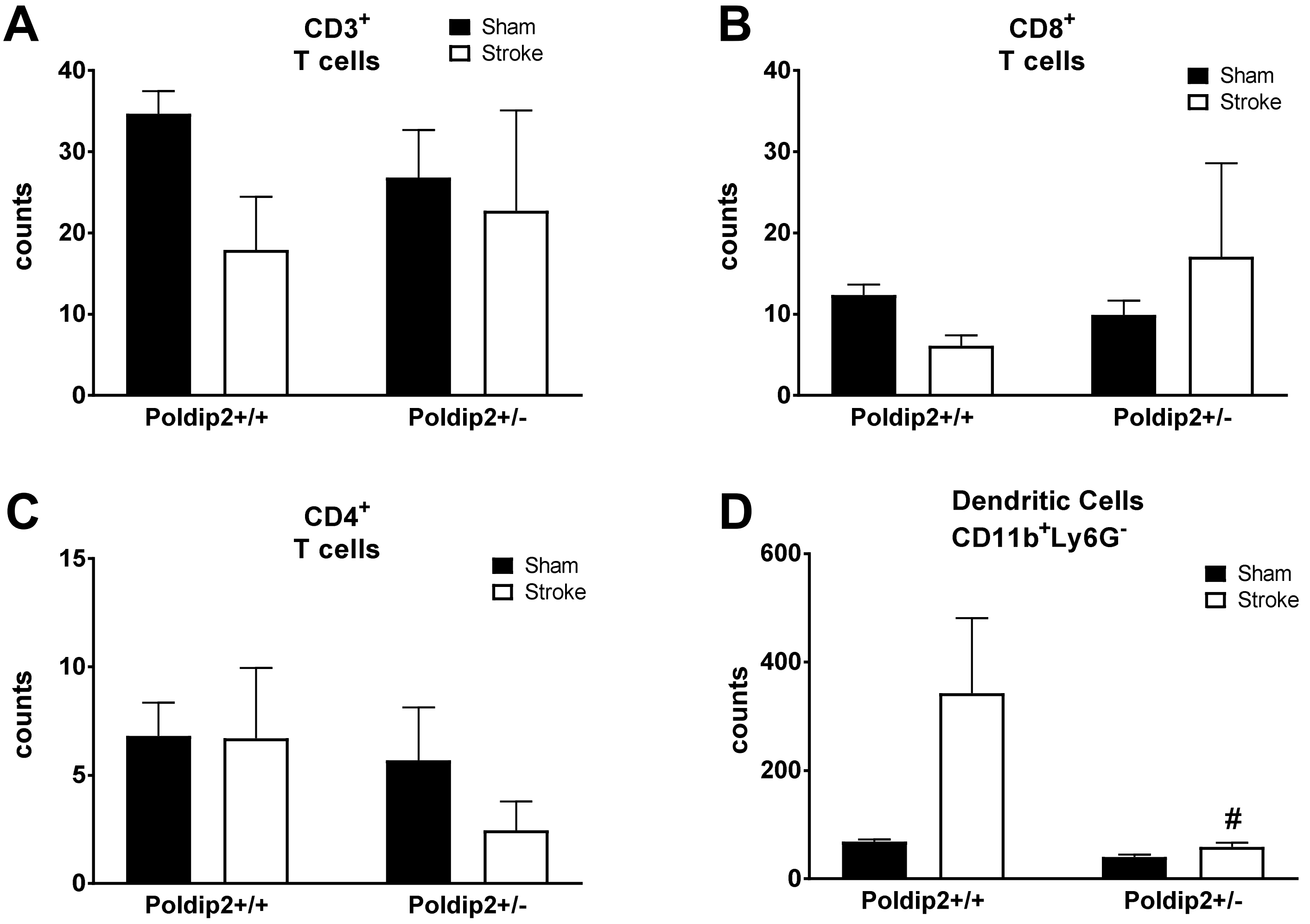

Supplemental Figure 3

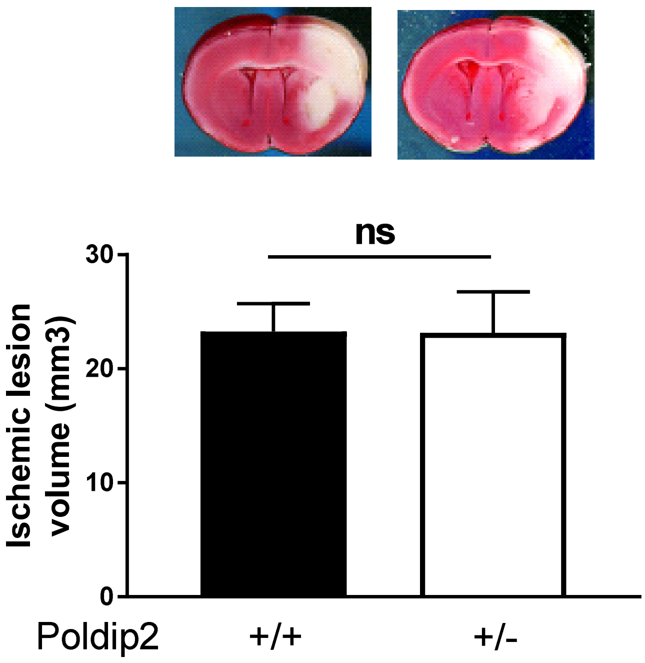

Supplemental Figure 4
(original western blots for Figure 3)

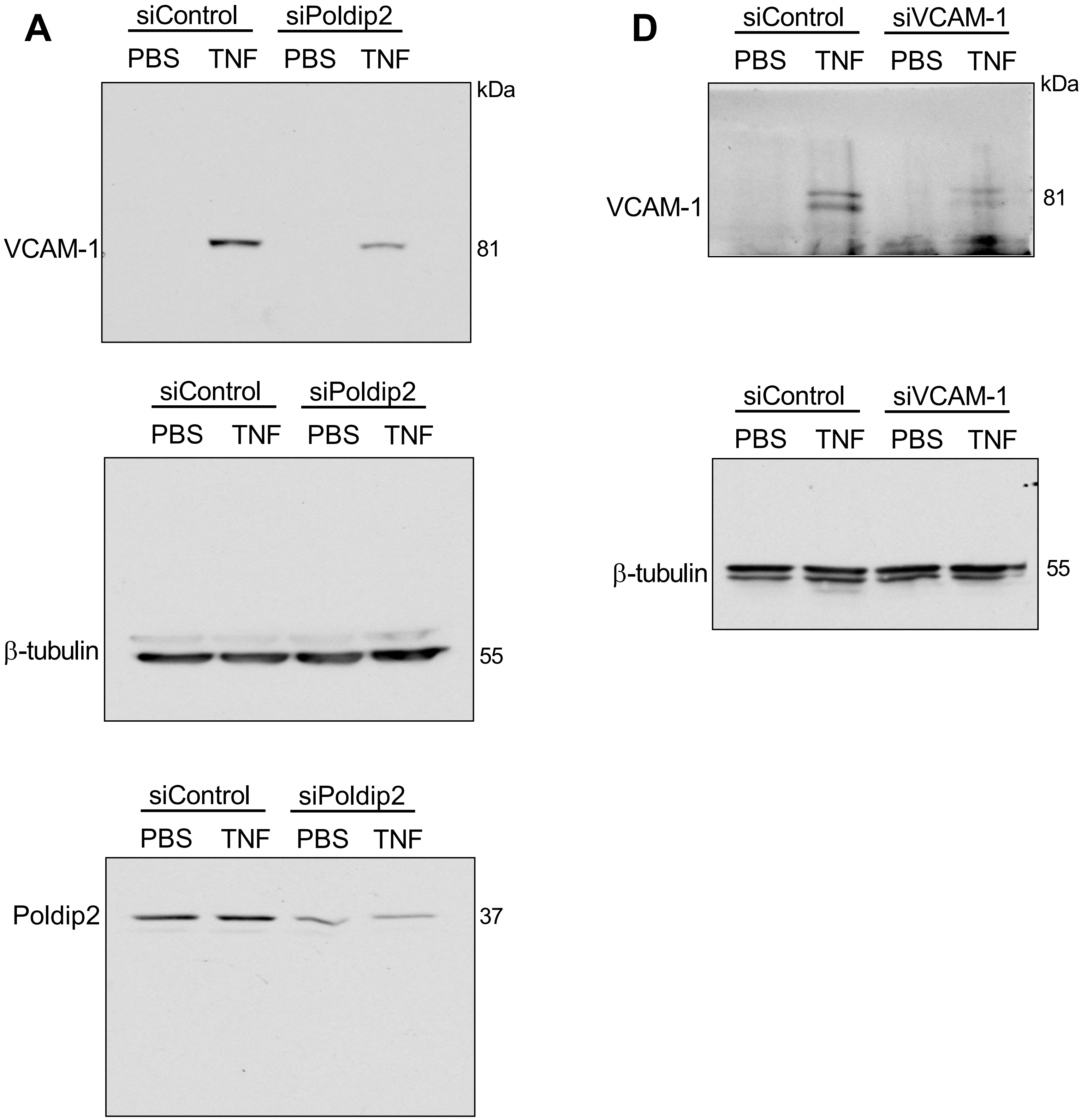

Supplemental Figure 5
(original western blots for Figure 4)

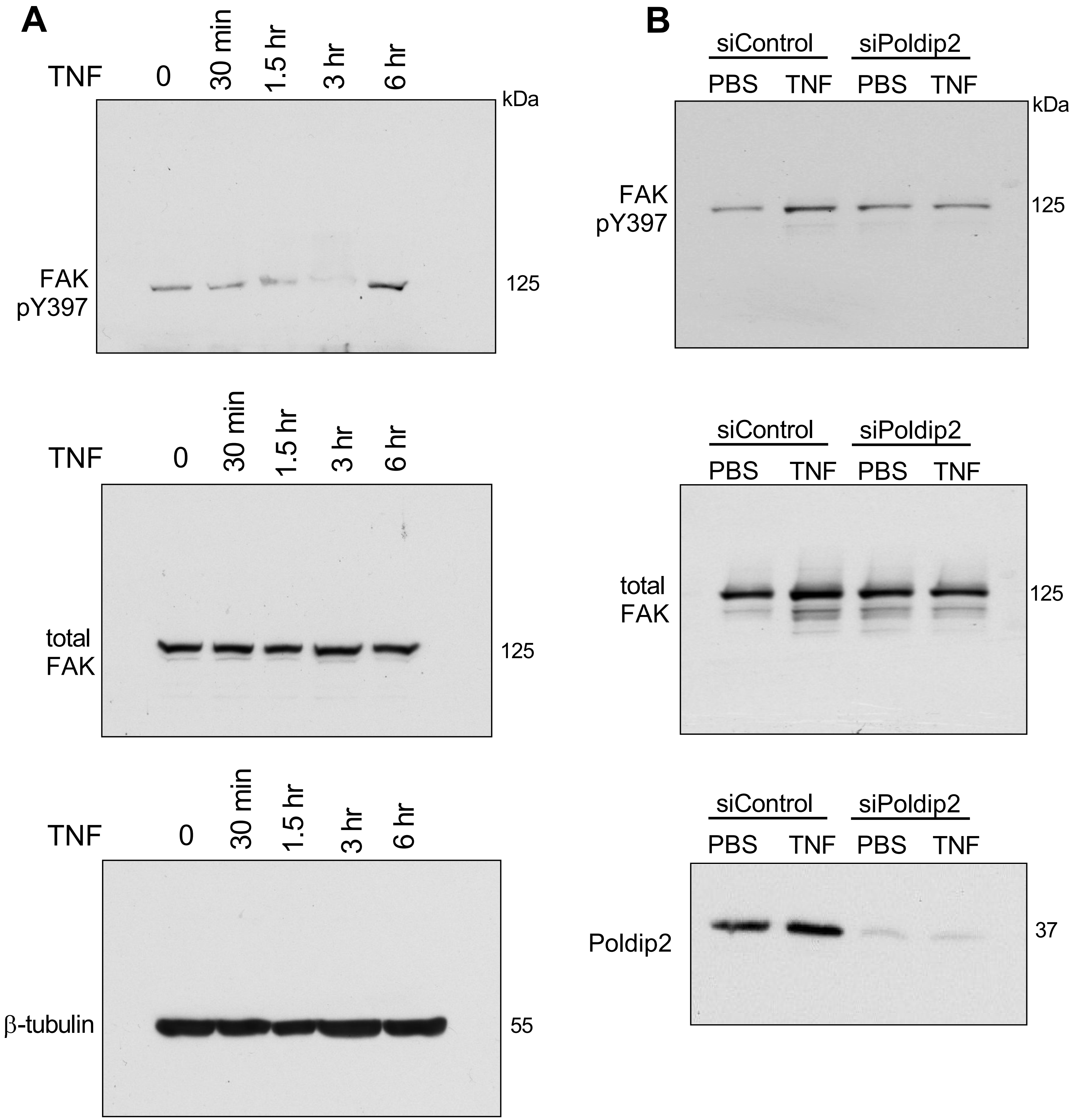

Supplemental Figure 6
(original western blots for Figure 5)

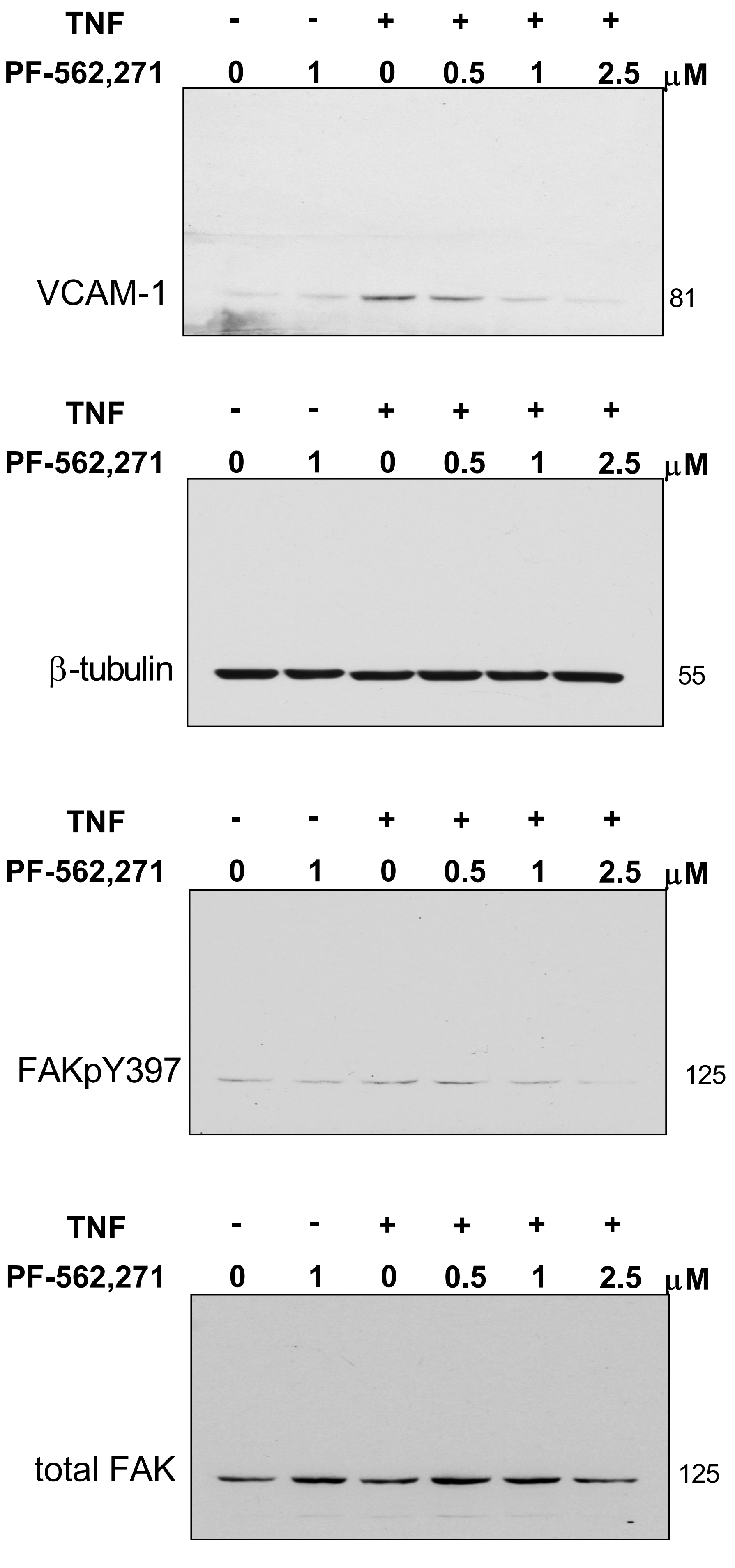

Supplemental Figure 7
(original western blots for Figure 6)

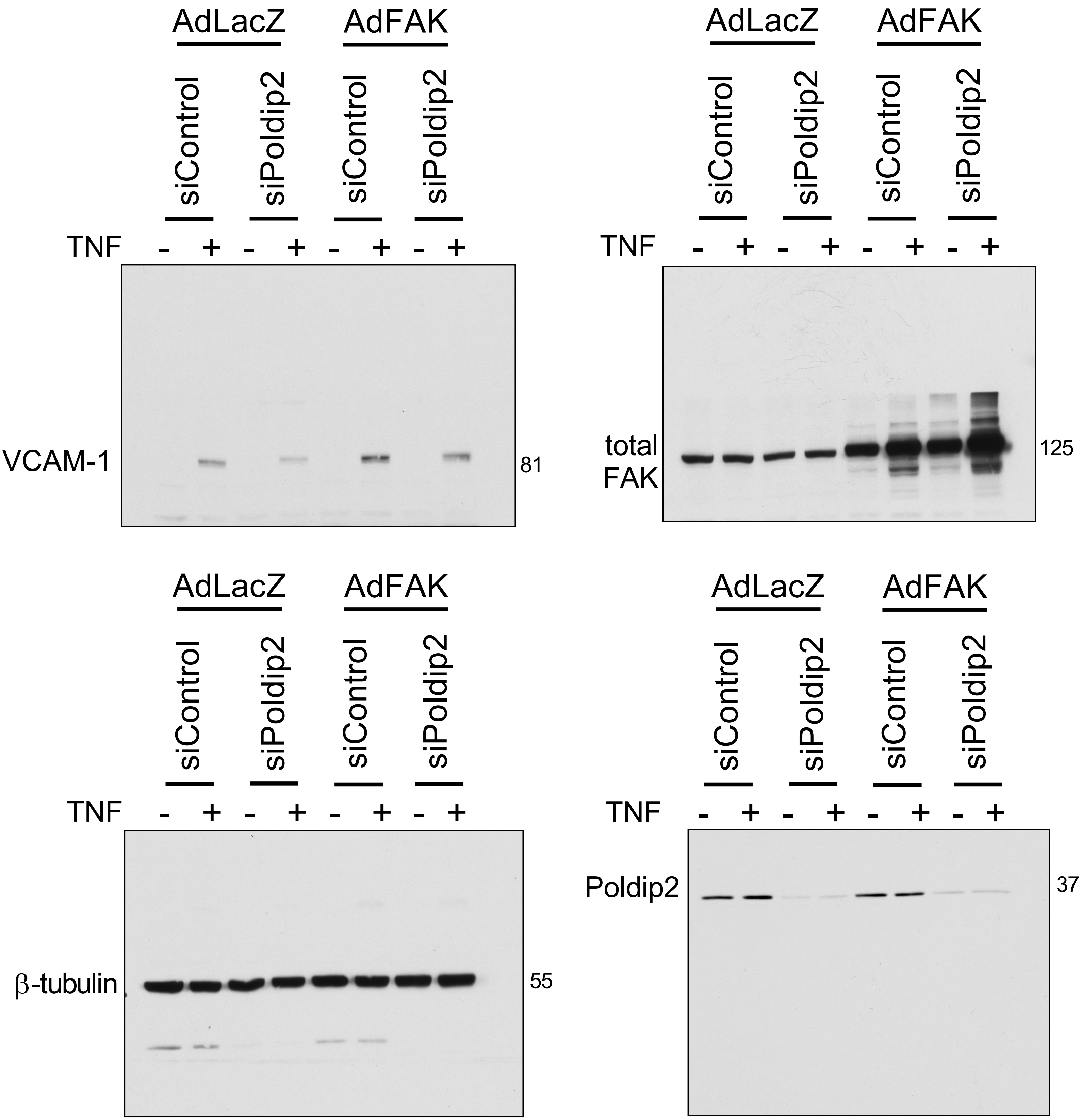

Supplement: Supplementary file 1 — Supplementary Information [file 41598_2021_84987_MOESM1_ESM.pdf]
